# IsoTV: processing and visualizing functional features of translated transcript isoforms

**DOI:** 10.1093/bioinformatics/btab103

**Published:** 2021-02-15

**Authors:** Siddharth Annaldasula, Martyna Gajos, Andreas Mayer

**Affiliations:** Otto-Warburg-Laboratory, Max Planck Institute for Molecular Genetics, Berlin 14195, Germany; Department of Mathematics and Computer Science, Freie Universität Berlin, Berlin 14195, Germany; Otto-Warburg-Laboratory, Max Planck Institute for Molecular Genetics, Berlin 14195, Germany; Department of Mathematics and Computer Science, Freie Universität Berlin, Berlin 14195, Germany; Otto-Warburg-Laboratory, Max Planck Institute for Molecular Genetics, Berlin 14195, Germany

## Abstract

**Summary:**

Despite the continuous discovery of new transcript isoforms, fueled by the recent increase in accessibility and accuracy of long-read RNA sequencing data, functional differences between isoforms originating from the same gene often remain obscure. To address this issue and enable researchers to assess potential functional consequences of transcript isoform variation on the proteome, we developed IsoTV. IsoTV is a versatile pipeline to process, predict and visualize the functional features of translated transcript isoforms. Attributes such as gene and isoform expression, transcript composition and functional features are summarized in an easy-to-interpret visualization. IsoTV is able to analyze a variety of data types from all eukaryotic organisms, including short- and long-read RNA-seq data. Using Oxford Nanopore long read data, we demonstrate that IsoTV facilitates the understanding of potential protein isoform function in different cancer cell types.

**Availability and implementation:**

IsoTV is available at https://github.molgen.mpg.de/MayerGroup/IsoTV, with the corresponding documentation at https://isotv.readthedocs.io/.

**Supplementary information:**

Supplementary data are available at *Bioinformatics* online.

## 1 Introduction

Alternative splicing diversifies transcriptomes of metazoans by generating transcript isoforms (Ule and Blencowe, 2019). Coding transcripts are then translated to protein isoforms, contributing to proteome diversity. The development of long-read sequencing technologies has enabled the characterization of full length transcripts and identification of non-canonical isoforms (Sakamoto *et al*., 2020). However, there is a lack of dedicated computational tools for protein isoform analysis and visualization, especially regarding novel isoforms.

Here, we introduce IsoTV (Isoform Transcript Visualizer), a versatile Snakemake (Koster and Rahmann, 2012) pipeline to analyze and visualize the functional features of translated transcript isoforms. IsoTV incorporates various tools to predict protein domains, secondary structure, disordered regions and post-translational modification sites. The visualization facilitates comparison of the set of transcript isoforms expressed across different conditions and helps to explore functional consequences of isoform differences. IsoTV supports a range of transcriptome sequencing technologies, including short-reads, and Oxford Nanopore (ONT) and Pacific Biosciences long-reads. In addition, the pipeline is able to process raw ONT long-reads to *de novo* assemble a transcriptome and quantify isoform expression. We demonstrate the functionality of IsoTV on cancer cell lines sequenced using ONT long-reads (The Singapore Nanopore Expression Consortium, 2020).

## 2 Materials and methods

IsoTV’s modular architecture allows visualization on a range of inputs containing at least a transcriptome FASTA file and the corresponding annotation GTF file, and a list of genes. If a file containing isoform expression is provided, gene and isoform expression can be compared across conditions.

IsoTV can process raw or basecalled ONT reads to *de novo* assemble the transcriptome. This sub-workflow is inspired by ONT’s long-read processing pipeline (Nanopore Technologies, 2019), with the following changes. If raw ONT signals are provided, the reads are basecalled with Guppy, and low quality reads are discarded using Filtlong (Wick, 2017). The transcriptome is then comprehensively defined for all samples.

Finally, Gffcompare (Pertea and Pertea, 2020) generates consensus sequences for all isoforms and removes redundant transcripts. After assembling the transcriptome, filtered reads are mapped (Li, 2018), quantified and normalized (Love *et al*., 2014). Supplementary Figure S1 illustrates the scheme of the pipeline.

An additional utility of IsoTV is its isoform translation approach. One obstacle of directly translating mRNA sequences is the presence of upstream open reading frames (uORFs), which are non-coding ORFs located upstream of the coding region (Zhang *et al*., 2019). Existing tools do not account for uORFs and rely on ribosome profiling data. We developed an algorithm that addresses both issues. Potential start codon sequences are scored using the position weight matrix of the motif for initiation of protein translation (Kozak, 1987), and the amino acid chain length is stored. The start sites’ score and their respective amino acid chain length are compared, and the most probable ORF determined from these criteria is translated.

**Fig. 1.**
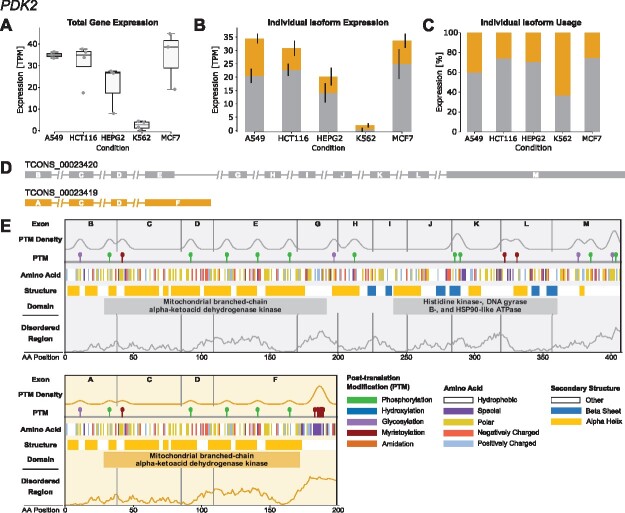
IsoTV visualization of translated transcript isoforms for *PDK2* in five human cancer cell lines using ONT data from the Singapore Nanopore Expression Project. For all sections, each isoform is consistently color-coded. (**A**) Total gene expression with each individual replicate plotted. (**B**) Individual isoform expression. (**C**) Isoform usage normalized to total gene expression. (**D**) Transcript composition. The lines represent introns, while the rectangles designate exons. Exons with identical 5′ and 3′ boundaries are annotated with the same identifier. (**E**) Feature plots of translated isoforms

The IsoTV visualization consists of three major sections depicting various facets of the analysis (Fig. 1). The first section illustrates the gene and isoform expression levels across all conditions (Fig. 1A–C). The second visualizes the intron-exon structure of all isoforms (Fig. 1D). The third summarizes the features of all translated isoforms, with each isoform having its own dedicated panel consisting of subsections that can be individually included (Fig. 1E). These sections and tools (de Castro *et al*., 2006; Cock *et al*., 2013; Erdős and Dosztányi, 2020; Torrisi*et al*., 2019) are further described in the IsoTV documentation.

## 3 Results

In order to evaluate the pipeline, we chose *ADAM15* because of its known increased expression in breast and lung cancers (Schütz*et al*., 2005) and well characterized alternative splicing events generating distinct transcript isoforms (Kleino*et al*., 2007). A detailed description of this case study is given in the Supplementary Material. Briefly, using basecalled ONT direct cDNA data for five human cancer cell lines (The Singapore Nanopore Expression Consortium, 2020), the annotation and expression plots from the visualization showed increased expression and usage for Isoform 3 that skips Exon W in breast and lung cancer cells (Supplementary Fig. S2A–D). Moreover, the feature plot for Isoform 3 lacks a proline-rich sequence and a disordered region compared with other isoforms (Supplementary Fig. S2E). This case study demonstrates that IsoTV is able to process ONT long-reads, identify transcript isoforms and characterize functional features of translated isoforms with intuitive visualizations.

## Supplementary Material

btab103_Supplementary_Data
