# Supplementary material for: IsoTV: processing and visualizing functional features of translated transcript isoforms
Source: Bioinformatics. 2021 Feb 15;37(18):3070–2. doi: 10.1093/bioinformatics/btab103 (PMC8479675; doi:10.1093/bioinformatics/btab103)
Supplement: btab103_Supplementary_Data [file btab103_supplementary_data.zip › Supplement_revised_Mayer.docx]

Supplementary Information to

IsoTV: processing and visualizing functional features of translated transcript isoforms

Siddharth Annaldasula^1,2^, Martyna Gajos^1,2^ and Andreas Mayer^1,*^

^1^Otto-Warburg-Laboratory, Max Planck Institute for Molecular Genetics, 14195 Berlin, Germany
^2^Department of Mathematics and Computer Science, Freie Universität Berlin, 14195, Berlin, Germany

-------------------------------------------------------------------------------------------------------------------------------

This document provides supplementary information to the accompanying paper. Complete IsoTV documentation can be found at <https://isotv.readthedocs.io/> and from <https://github.molgen.mpg.de/MayerGroup/IsoTV>.

**Table of Contents**

[A. Supplementary Figure 1 2](#_Toc63419762)

[B. Supplementary Figure 2 3](#_Toc63419763)

[B.1 Results Extended 4](#_Toc63419764)

[References 5](#_Toc63419765)

# A. Supplementary Figure 1

Supplementary Figure 1. Scheme for the IsoTV pipeline. Red circles highlight possible starting locations for the pipeline, either with raw ONT signal or processed transcriptome file.

# B. Supplementary Figure 2

Supplementary Figure 2. IsoTV visualization of transcript and translated transcript isoforms for *ADAM15* in five human cancer cell lines using ONT data from the Singapore Nanopore Expression Project. (A) Total gene expression with each individual replicate plotted. (B) Individual isoform expression. Isoform 3 is marked in A549 and MCF7. (C) Isoform usage normalized to total gene expression. Isoform 3 is marked in A549 and MCF7. (D) Transcript composition. The region spanning the alternatively skipped exons in selected isoforms are marked with a red dashed box. (E) Zoomed in feature plots of selected translated isoform regions marked by the red dashed box in (D). Proline is classified as a special amino acid.

## B.1 Results Extended

In order to evaluate the pipeline, we chose *ADAM15* because of its known increased expression in breast and lung cancers (Schütz et al., 2005) and well characterized alternative splicing events generating distinct transcript isoforms (Kleino et al., 2007). Experimental evidence has further shown proline-rich sequences encoded by the last few exons, serving as a binding site for SH3 domain-containing proteins to localize tight junction proteins to the cell membrane (Mattern et al., 2019). However, one isoform skips ‘Exon 19’, which is essential for SH3 binding site formation. To validate these results, basecalled ONT direct cDNA data was used for five human cancer cell lines: A549 (lung), HCT116 (colon), HepG2 (liver), K562 (blood), and MCF7 (breast) (https://github.com/GoekeLab/sg-nex-data, The Singapore Nanopore Expression Consortium, 2020). Annotation and expression plots from the visualization showed increased expression and usage for Isoform 3 of *ADAM15* that skips exon W (experimentally annotated ‘Exon 19’) in A549 (lung) and MCF7 (breast) samples compared to the other samples (Supp. Fig. 2A-D). Moreover, the feature plot for Isoform 3 lacks a proline-rich sequence and a disordered region compared with other isoforms (Supp. Fig. 2E). This case study demonstrates that IsoTV is able to process ONT long-reads, identify transcript isoforms, and characterize functional features of translated isoforms with intuitive visualizations.

# References

Kleino,I. et al. (2007) *ADAM15* gene structure and differential alternative exon use in human tissues. BMC Mol. Biol., 8, 90.

Mattern,J. et al. (2019) ADAM15 mediates upregulation of Claudin-1 expression in breast cancer cells. Sci. Rep., 9, 12540.

Schütz,A. et al. (2005) Expression of *ADAM15* in lung carcinomas. Virchows Arch., 446, 421–429.

The Singapore Nanopore Expression Consortium (2020) The Singapore Nanopore Expression Project (SG-NEx) data pre-release v0.1. Zenodo. http://doi.org/10.5281/zenodo.4159715
